# The interplay between academic performance and quality of life among preclinical students

**DOI:** 10.1186/s12909-015-0476-1

**Published:** 2015-10-31

**Authors:** Mohammad Abrar Shareef, Abdulhadi A. AlAmodi, Abdulrahman A. Al-Khateeb, Zainab Abudan, Mohammed A. Alkhani, Sanderlla I. Zebian, Ahmed S. Qannita, Mariam J. Tabrizi

**Affiliations:** 1 St. Vincent Mercy Medical Center, 2213 Cherry Street, Toledo, OH 43608-2691 USA; 2 University of Mississippi Medical Center, Jackson, MS USA; 3 Alfaisal University, Riyadh, Saudi Arabia

## Abstract

**Background:**

The high academic performance of medical students greatly influences their professional competence in long term career. Meanwhile, medical students greatly demand procuring a good quality of life that can help them sustain their medical career. This study examines validity and reliability of the tool among preclinical students and testifies the influence of their scholastic performance along with gender and academic year on their quality of life.

**Methods:**

A cross sectional study was conducted by distributing World Health Organization Quality of Life, WHOQOL-BREF, survey among medical students of year one to three at Alfaisal University. For validity, item discriminate validity(IDV) and confirmatory factor analysis were measured and for reliability, Cronbach’s α test and internal item consistency(IIC) were examined. The association of GPA, gender and academic year with all major domains was drawn using Pearson’s correlation, independent samples *t*-test and one-way ANOVA, respectively.

**Results:**

A total of 335 preclinical students have responded to this questionnaire. The construct has demonstrated an adequate validity and good reliability. The high academic performance of students positively correlated with physical (*r* = 0.23, *p* < 0.001), psychological health (*r* = 0.29, *p* < 0.001), social relations (*r* = 0.11, *p* = 0.03) and environment (*r* = 0.23, *p* < 0.001). Male student scored higher than female peers in physical and psychological health.

**Discussion:**

This study has identified a direct relationship between the academic performance of preclinical students and their quality of life.

**Conclusion:**

The WHOQOL-BREF is a valid and reliable tool among preclinical students and the positive direction of high academic performance with greater QOL suggests that academic achievers procure higher satisfaction and poor achievers need a special attention for the improvement of their quality of life.

## Background

The term, quality of life, was first used in US after the Second World War, to demonstrate that having a good life is of more value than just being financially well [1, 2]. As the term became more widely accepted and used, a panel of definitions emerged from different studies with some consensus about its possible use in exchange for the term “overall satisfaction” [3]. The World Health Organization (WHO) has then defined the quality of life as, “*an individual’s perception of their position in life in the context of the culture and value systems in which they live and in relation to their goals, expectations, standards and concerns*” [4]. This definition entails a mean of portraying various aspects of an individual’s lifestyle which can reflect on his/her overall life satisfaction [1, 5].

Following that, the WHO started studying and examining various instruments that can possess a greater validity and reliability with the highest possible accuracy of measuring quality of life. The WHO-QOL 100-item survey was the first to be introduced but required some rectifications and reforming of some of its domains [6]. An abbreviated version, WHOQOL-BREF, was then released as a self-administered questionnaire comprising of 26 items for the assessment of four major domains including physical health, psychological health, social relations and environment [7, 8]. Later, this vastly-applied, cross-cultural and short form construct was translated into more than 40 languages, gained its validity and reliability from studies at different countries and became the standard subjective measurement of the quality of life [8–14]. Among Arab countries, a study from Kuwait has already explored and reported its validity and reliability [11].

Exploring medical students’ quality of life became increasingly important as they encounter a variety of stressors in the college including heavy study loads and stressful exams [15]. As medical students face these impediments throughout their study in the college mainly during the preclinical years, acquiring higher academic and personal achievements becomes more challenging [16]. Previous studies have elucidated noticing a dramatic reduction of medical students’ quality of life when they start encountering patients during the start of their clinical years [10, 17]. Therefore, it would be of interest to contemplate preclinical students’ quality of life and investigate various factors that can influence it, aiming for a positive direction in their quality of life.

The academic performance of medical students is measured as Grade Point Average (GPA). A longitudinal study reported that the academic performance of medical students predicts their professional competence in their medical career [18]. Hence, the high academic performance of preclinical students can result in an uplift of their professional competence in the clinical phase, where encountering patients in professional manner becomes very crucial.

While the quality of life of medical students has been studied [1, 9], studies regarding the correlation of academic achievement of preclinical students with their quality of life are still lacking. Thus, the prime aims of this study include: 1) To measure the validity and reliability of the tool, WHOQOL-BREF survey, among preclinical students at Alfaisal University, 2) To draw a correlation between their academic performance and their quality of life, and 3) To ascertain the role of other concomitant factors like gender and academic year on students’ self evaluation of their quality of life.

## Methods

### Study setting and population

The medical curriculum at Alfaisal University enlightens students with basic medical knowledge in the first three years and they are considered to be in preclinical phase. The following two years are when students spend most of their time in the hospital in different rotations assigned to them. The medical curriculum is English-based and students are expected to show good level of competence in English prior to admission to the college. The medical curriculum is delivered in an integrated fashion and preclinical students are exposed to both problem-based and team-based learning curriculum.

The GPA of students are grouped into four levels: 1) below average students who tend to have GPA of lower than 2.5 and they usually face difficulties stepping over and passing the required courses, 2) average students with GPA between 2.5 and 3.0, 3) good students whose GPA is of 3.0–3.5, and 4) honor students who have their GPA between 3.5 and 4.0.

In order to mitigate the influence of encountering patients on quality of life of medical students, the study population entailed preclinical students, who have not exposed to patients yet. The total number of preclinical students who were targeted in this study was 561 students; 226 from first year, 176 from second and 159 from third year.

### Development of study instrument

The WHOQOL-BREF survey is a widely accepted cross-cultural survey that was translated into many languages in various developed and developing countries [8–14]. This cross sectional study utilized the use of two questionnaires that were placed on the same web page and distributed anonymously to all preclinical students via their Alfaisal emails. The first questionnaire contained demographic characteristics such as the GPA, gender and academic year and the second one was the Standard English version of WHOQOL-BREF that was modified to 22 items. Each question had certain response options and these options were followed by 5-point Likert scale and ranged from (very poor/not at all/very dissatisfied/never) to (very good/completely/very satisfied/always). The questionnaire targeted four main domains and the score of each domain were transformed into linear scale that ranged 0–100. The number of items underneath each domain was five items for physical health, seven for psychological health, three for social relations, and seven for environment.

### Statistical analysis

The analysis of the data was processed in multiple steps. First, the frequency distribution of the demographics was determined. Next, the item discriminate validity (IDV) was explored via assessing if Pearson’s correlation coefficient of each item with its respective domain is higher than other domains. The factorability of the data was then assessed utilizing Kaiser-Meyer-Olkin Measure of Sampling Adequacy test (KMO) and Barlett’s Test of Sphericity. The confirmatory factor analysis was then processed by structuring the four-factor model. Next, the internal item consistency (IIC) was extrapolated with the correlation requirement of more than >0.4 of each item with its domain [17, 19, 20]. Furthermore, Cronbach’s α coefficient test was used to examine the extent of internal uniformity among the tested domains.

The Pearson’s correlation was also used to determine the correlation of the academic performance using GPA with four domains along with each item underlying it. The independent sample *t*-test and one-way Analysis of Variance (One way-ANOVA) examined the gender and academic year-specific difference in students’ evaluation of each domain. All the analyses were carried out using IBM SPSS statistical software version 20 except for the confirmatory factor analysis which was done using AMOS software version 21.

### Ethical consideration

The study was approved by the Institutional Review Board at Alfaisal University. All students were informed about the purpose and aim of the study, and filled the survey voluntarily. No specific identification questions were used; hence, the results remain confidential and are only used for the purpose of the study.

## Results

### Demographic characteristics of students in the study

The total number of students who responded to the survey was 335 students with the response rate of 60 % and among them, 46 % were male and 44 % were from the female side. Around 36 % of students were from first year (*n* = 119) while others were from second and third years. Most of the students had GPA of higher than 3.5 (41 %, *n* = 139). Students’ demographic characteristics are shown in Table 1.

**Table 1:** Demographic characteristics of study subjects and their scores in each domain

Variables	*N* (%)	Physical health	Psychological health	Social relations	Environment
		X + *s*	X + *s*	X + *s*	X + *s*
GPA					
Above 3.5	139 (41 %)	58.54 + 18.30	60.53 + 15.18	68.65 + 20.15	66.56 + 13.50
3.0 – 3.49	120 (36 %)	53.27 + 21.02	55.83 + 17.74	66.73 + 24.91	63.92 + 17.75
2.5 – 2.99	45 (13 %)	49.51 + 18.52	51.09 + 18.23	66.04 + 26.33	58.71 + 19.14
Below 2.5	31 (9 %)	43.10 + 19.01	43.48 + 16.39	58.29 + 23.72	54.32 + 17.51
Gender					
Male	154 (46 %)	57.27 + 20.00	58.26 + 16.88	66.23 + 25.07	65.48 + 17.29
Female	181 (44 %)	51.24 + 19.43	54.08 + 17.59	67.02 + 21.58	61.68 + 15.98
Academic year					
First year	119 (36 %)	56.19 + 19.30	58.40 + 17.62	67.08 + 24.83	63.64 + 15.71
Second year	115 (34 %)	52.03 + 20.00	54.77 + 16.45	65.84 + 23.59	62.97 + 16.69
Third year	101 (30 %)	53.69 + 20.40	54.58 + 17.94	67.07 + 20.90	63.69 + 17.89

### The instrument validity

Each item underneath the four domains is well-correlated with its respective domain than others. This indicates that the construct reflects adequacy in overall IDV as shown in Table 2. Results of the KMO test was 0.92, with a Barlett’s Test of Sphericity *P* value of <0.001. The confirmatory factor analysis revealed an adequate fit to four-factor model when two matching covariances were allowed to be correlated. For instance, the majority of retrieved conditions meet the criteria for model fitness including the *X*^2^ = 380, root mean square error of approximation (RMSEA) = 0.062; the comparative fit index (CFI) = 0.923 and Tucker-Lewis coefficient (TLI) = 0.90, the root mean square residual (RMR) = 0.06, the goodness-of-fit index (GFI) = 0.90 and the adjusted goodness-of-fit index (AGFI) = 0.87 [21]. All item loadings were greater than 0.40 and are demonstrated in Table 3 [17, 19, 20]. The results of the analysis are suggestive of an acceptable validity of the construct. All domains were positively correlated among each other and the exact correlation coefficients are shown in Table 4.

**Table 2:** Analysis of item discriminate validity and internal item consistency of the construct

Domain items	Physical health	Psychological health	Social relations	Environment
Physical health				
Energy	.754^a^	.552	.396	.526
Mobility	.590^a^	.498	.418	.448
Sleep and rest	.725^a^	.433	.357	.432
Activities of daily living	.860^a^	.608	.419	.525
Work capacity	.826^a^	.598	.474	.497
Psychological health				
Spirituality	.521	.650^a^	.415	.505
Self-esteem	.411	.704^a^	.401	.331
Thinking, learning, memory and concentration	.544	.613^a^	.273	.339
Personal beliefs	.302	.570^a^	.208	.234
Bodily image and appearance	.458	.653^a^	.415	.430
Positive feelings	.606	.774^a^	.585	.497
Negative feeling	.463	.658^a^	.397	.364
Social relations				
Personal relationships	.517	.591	.882^a^	.558
Social acceptance	.456	.502	.819^a^	.514
Social support	.423	.405	.857^a^	.591
Environment				
Physical safety	.410	.479	.464	.617^a^
Physical environment	.386	.328	.243	.553^a^
Financial resources	.387	.417	.494	.689^a^
Opportunities for acquiring new information	.384	.352	.392	.614^a^
Leisure activities	.457	.376	.277	.531^a^
Home environment	.445	.363	.524	.731^a^
Accessibility of health care	.438	.358	.516	.757^a^

^a^Both IDV (higher correlation with its respective domain) and IIC (correlation of more than 0.4) are achieved

**Table 3:** Results of confirmatory factor analysis and multivariate regression analysis of each study item

Domain items	Factor 1	Factor 2	Factor 3	Factor 4
Physical health				
Energy	0.70			
Mobility	0.56			
Sleep and rest	0.59			
Activities of daily living	0.85			
Work capacity	0.78			
Psychological health				
Spirituality		0.62		
Self-esteem		0.58		
Thinking, learning, memory and concentration		0.55		
Personal beliefs		0.38		
Bodily image and appearance		0.57		
Positive feelings		0.81		
Negative feeling		0.57		
Social relations				
Personal relationships			0.84	
Social acceptance			0.73	
Social support			0.75	
Environment				
Physical safety				0.56
Physical environment				0.41
Financial resources				0.61
Opportunities for acquiring new information				0.53
Leisure activities				0.46
Home environment				0.66
Accessibility of health care				0.67

**Table 4:** Correlations among the four major domains in the study

	Physical	Psychological	Social	Environment
Physical	1			
Psychological	0.71*	1		
Social	0.64*	0.59*	1	
Environment	0.78*	0.59*	0.65*	1

*P < 0.0001

### The reliability of the questionnaire

All the 22 items measured in the study revealed that correlation of each item with its respective domain is higher than 0.40. In fact, the lowest correlation coefficient encountered in the study is 0.53, indicating strong internal item consistency (Table 2).

Moreover, the results of Cronbach’s α coefficient test include 0.81 for physical health, 0.78 for psychological health, 0.81 for social relations, and 0.78 for environment. The overall Cronbach’s α test is 0.80 suggesting a good internal uniformity of the tested domains.

### Difference of students’ academic performance based on their gender and academic year

Both male and female students have similar academic performance (*p* = 0.97). In addition, the academic year of students did not extrapolate any difference in their GPA (*p* = 0.11).

### Students’ self ratings in physical health

**Fig. 1:**
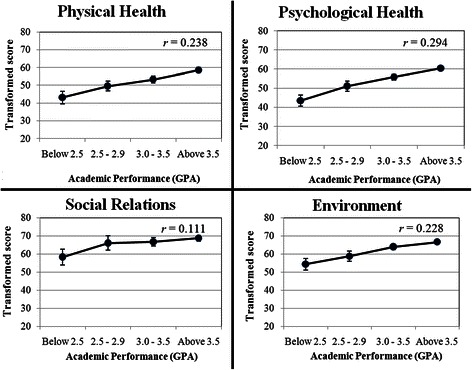
Pearson’s correlation of GPA with all four domains. *r* is correlation coefficient

The academic performance of students is positively correlated with their overall physical health status (*r* = 0.238 & *p* < 0.001) (Fig. 1). More specifically, an increase in students’ GPA is associated with a rise in their energy (*r* = 0.20 & *p* < 0.001), mobility (*r* = 0.16 & *p* = 0.004), activities of daily living (*r* = 0.21 & *p* < 0.001) and work capacity (*r* = 0.26 & *p* < 0.001). In addition, the male gender scored higher in this domain compared to their female peers (*p* = 0.007). However, the academic year of the students did not depict any significant effect (*p* = 0.28).

### Students’ self-assessment of psychological health

The high GPA of students is found to be associated with high scoring in psychological health (*r* = 0.294 & *p* < 0.001) (Fig. 1). For instance, obtaining a good GPA correlates positively with high scoring in spirituality (*r* = 0.18 & *p* = 0.001), self-esteem (*r* = 0.16 & *p* = 0.003), thinking, learning, memory and concentration (*r* = 0.24 & *p* < 0.001), personal beliefs (*r* = 0.15 & *p* = 0.005), bodily image and appearance (*r* = 0.13 & *p* < 0.015), positive feelings (*r* = 0.27 & *p* < 0.001) and less negative feelings (*r* = 0.20 & *p* < 0.001). Furthermore, male students were noted to have a higher rating in this domain compared to female students (*p* = 0.021). The academic year, on the other hand, did not demonstrate any effect (*p* = 0.18).

### Students’ self evaluation of social relations

The academic performance of students correlates with an incline in their scoring in social relations (*r* = 0.11, *p* = 0.03) (Fig. 1). In fact, personal relationships (*r* = 0.1 & *p* = 0.013) and social support (*r* = 0.11 & *p* = 0.038) are correlated with academic performance of preclinical students.

Both gender and the academic year did not alter students’ ratings in this domain with the *p* values of 0.72 and 0.85, respectively.

### Students’ self-ratings of their surrounding environment

In regards to the relationship of the GPA with students’ self-evaluation of their environment, the GPA is still considered to be correlated with their scores (*r* = 0.23, *p* < 0.001) (Fig. 1). For instance, honor students with high GPA tend to have a better ranking in physical safety (*r* = 0.20 & *p* < 0.001), physical environment (*r* = 0.12 & *p* = 0.024), financial resources (*r* = 0.21 & *p* < 0.001), opportunities for acquiring new information (*r* = 0.20 & *p* < 0.001), home environment (*r* = 0.21 & *p* < 0.001) and accessibility of health care (*r* = 0.11 & *p* = 0.037).

Similar to the previous domain, both gander and academic year-based differences in students’ ratings in this domain were not significant (*p* = 0.27 & *p* = 0.98).

## Discussion

In concordance with the previous studies that validated this construct, the current study revealed an acceptable validity and reliability of the gauge among preclinical students at Alfaisal University, which makes it suitable for the analysis of factors that can be associated with the measurable outcomes. Our study demonstrated that all domains are positively correlated to each other, which signifies that students’ ratings in each domain were very similar to their scores in other domains. This is consistent with the finding of WHO-QOL study from Iran where medical students addressed a similar satisfaction in all the assessed components [1]. Another study compared the QOL of medical students with the general population and found that medical students scored lower in physical health, psychological health and environment but not in social relations [9]. Similarly, this study entailed the highest rating in social relations where the students reported a positive attitude towards their interactions with their friends and the support they provide to each other. This is a remark of a positive implication for their future as physicians are in necessity of embracing this friendly character in order to polish their doctor-patient relationship [22]. On the other hand, the physical health had the lowest rank among other domains and the “sleep and rest” component attained the lowest rating than other physical health items. For instance, as medical students comply with their large academic load, many of them do not devote much time for rest or sleep especially when it is close to their exams [23].

The academic performance of medical students has shown to be a positive predictor for their developed professional competence in their long term career [18]. In addition, examining the association of academic performance and other factors with students’ self-scoring of physical, psychological health, social life and environment is essential. In this study, students with higher academic performance scored higher in all domains and male students are better than female students in physical and psychological health domains. The previous international data about the academic performance of medical students based on their genders is varied, where mostly reported a better performance of female students [24, 25] and one study reported no difference [26]. A study from a local university revealed that females procured a higher GPA than male students [27]. Upon examining the relationship between the two demographic factors in this study; gender and GPA, results suggest a lack of GPA difference between male and female students. Hence, both gender and GPA are considered independent correlates with the domains.

Students who devote more time for their academia tend to score better in physical health than those who merely pass. Previous studies deduced that physical health and the academic performance of students run in parallel [28]. A study among undergraduate students in US revealed that students with high GPA are more engaged in physical health in comparison to their peers with low academic outcomes [29]. However, a study on college of science graduates extrapolated a lack of significant effect of physical health on GPA [30]. The latter finding is supported by another study that claimed for an existence of other potential influences that can strongly impact the GPA; like hard-work and commitment, both of which can escalate the academic performance [31]. Our data suggests that as students move from one level of GPA to a higher one, an associative increase of around 5 % is noted in the physical health domain (Table 1). A possible explanation for this stratified increment is that energetic students tend to spend ample time studying the provided materials and their effort pays back in exams. Also, the high energy in these students may engage them into other daily living activities and may also provide them a greater capacity to work in their field of interest. On the other hand, below average students face difficulties in involving in other activities as their academic performance negatively impacts their energy and makes them less interested for taking part in other day-to-day activities [29]. Despite the lack of a significant correlation of the sleep with the GPA in the current study, a previous date averted that high academic achievers sleep longer (>9 h) than those with less academic performance. This could be due to the proper time management by students with high GPA, allowing them to go to bed earlier and wake up early and on-time [32].

A wide range of studies have ascertained the effect of scholastic performance on various components of psychological health including emotional intelligence, anxiety and depression [33, 34]. Still, very few studies investigated the items that belong to this domain in the short version of WHO-QOL survey. In this study, gaining five points in psychological health is associated with one level increment in the GPA. In order to testify this effect, each item belongs to this domain was assessed and results showed that all items were positively correlated with the academic performance. Several studies reported that an inflation of GPA was attributed to an incline in the level of spirituality, intelligence, motivation and self-esteem, and a decline in depression [35, 36]. As the interplay among these components becomes coherent and consolidated, one shall feel flourishing of his/her psychological health. Self-attainment of these components is heavily required by medical students as they will demand spirituality, motivation, positive feelings and self-esteem to deal with long working hours in their future clinical endeavors. Thus, an excellent academic achievement is associated with fulfilling highly demanding components necessary for the career prosperity of future doctors.

In regards to social relations, medical students did not explicitly demonstrate any difference in scoring this domain when compared to general population as discussed before [9]. Even though the overall high scoring of this section is associated with high GPA, a clear difference in GPA can be noted among below average students in comparison to other groups but not between excellent, good and average students (Table 1). As this domain assesses social support and personal relationships of preclinical students, both of these items are enormously essential for their future clinical endeavor. As high achievers in the academia seem to spend ample time in improving their scholastic outcome, they also tend to have a contextual enhancement in their e and social interaction skills.

The matriculation of physical activities in medical students’ life may be considered a mean for possessing an improvement in their environment. Many studies have enlightened the relationship between the scholastic achievements and physical environment where high achievers are more prone to impose a positive remark on their physical activity [29]. The current data coincides with these studies in regards to the positive correlation of GPA with preclinical students’ surrounding environments. Certain items from psychological health; such as self-esteem, also contribute to the physical activity of college students [36]. The financial issue, in turn, is one of the commonest concerns for medical students worldwide [37]. The system at Alfaisal University allows students to apply for merit-based or need-based scholarships in order to cover part of their tuition fees. The rest of the fees along with students’ daily expenses are usually covered by their parents. There might be two possible rationales behind the phenomenon of honor students entailing more financial support in this study. First, as long the parents start feeling a positive progress of their sibling in his/her academia, they may provide more financial support as an amicable award for his/her hard-work. Secondly, the merit-based scholarships are offered to honor students which, in turn, relieve the burden of student-loans for such preclinical students. Despite the latter rationale is seemingly more acceptable, both are imperative implication for offering a better environment for medical students.

The gender-specific effect on scores of physical and psychological health domains was noted. Similar to the current study, previous studies have demonstrated a higher rating of male students in physical and psychological health than the female counterparts [10, 38] and the proposed rational behind this is that females are more sensitive to pressure than their male peers [39]. A local study evaluated the gender based difference in physical activity among adults and deduced that females’ contribution in physical activity and exercise is significantly lower than their male peers [40]. There are limited studies tackling the change of quality of life across the preclinical years. All these factors can play an influential role in reducing the quality of life of female preclinical students.

There are limited studies tackling the change of quality of life across the preclinical years. The current study did not depict any difference in students’ ratings in the four domains across different years. A previous study from China reported that there was no difference in physical, psychological health and environment among the preclinical students in year 1 and 2, since year 3 students are already in clinical phase as per their curriculum. Socials relations, on the other hand, was the only domain that was rated less by second year students and this was due to the heavy workload that Chinese students have prior to the start of clinical phase. As the preclinical phase at Alfaisal University is covered over three years, covering the materials over this amount of period might mitigate the difference reported in the previous study [10].

As medical colleges strive to provide the optimal learning environment to students, more attention needs to be directed towards consistent measurement of students’ quality of life. Medical schools should build reforms in medical education and provide recreation centers in order to minimize the stress and burnout of students. It should also provide a positive environment and greater support for preclinical students who are poor in their academic performance. This can be achieved by establishing counseling facilities that can serve those with physical and psychological difficulties, aiming for a positive change towards their quality of life especially before the preclinical phase. Preclinical students, on the other hand, have to identify the impediments they face and seek for an advice from the faculty in order to find solutions for it. Furthermore, preclinical students who encounter difficulties in progressing in their academia should also ask for help and appeal for a positive change.

The main limitations of the study include; 1) The sample size of the study was drawn from one private institution where students and their parents may be financially stressed, in addition to students functioning in a very competitive setting, 2) The weak correlation could be due to the presence of other factors that were not examined including confidence in career development, students’ hometown location and interest in area of studies, and these factors need to be taken into account in future studies [10]. Finally, 3) not all students are exposed to same facilities and they might have some other academic factors that may influence their quality of life.

## Conclusion

The WHOQOL-BREF has shown to be a valid and reliable tool in assessing the quality of life of preclinical students at Alfaisal University. The academic performance of students has positively correlated with their quality of life. Male students demonstrated higher quality of life compared to their female peers, without any noticeable effect of academic year. Medical schools need to offer solutions for poor achievers before starting the clinical phase and these students should seek help from their advisors to overcome the obstacles.
